# Comparative evaluation of root dentin thickness following hand and rotary instrumentation of primary molar root canals using cone-beam computed tomography - an invitro study

**DOI:** 10.1186/s12903-026-09005-y

**Published:** 2026-06-26

**Authors:** Rincy M Thomas, Rashmi Nayak, Medhini Madi, Ravindranath Vineetha

**Affiliations:** 1 Rincy M Thomas, Department of Paediatric and Preventive Dentistry, Manipal College of Dental Sciences, Manipal Academy of Higher Education, Manipal, 576104 Karnataka India; 2 Department of Paediatric and Preventive Dentistry, Manipal College of Dental Sciences, Manipal Academy of Higher Education, Manipal, 576104 Karnataka India; 3 Department of Oral Medicine and Radiology, Manipal College of Dental Sciences, Manipal Academy of Higher Education, Manipal, 576104 Karnataka India

**Keywords:** Cone Beam Computed Tomography, Root dentin, Endodontics, Primary teeth

## Abstract

**Background:**

Remaining Root Dentin Thickness (RRDT) after root canal instrumentation is an important parameter to be assessed with newer file systems. This study aimed to evaluate the root dentin thickness following instrumentation of primary molar root canals using manual or pediatric rotary file systems.

**Methods:**

Fifty-one extracted human primary molars were prepared with access cavity preparation and working length determination. Preliminary Cone Beam Computed Tomography (CBCT) images were obtained with specimens mounted on vinyl polysiloxane templates. Root dentin thickness was measured at three predetermined levels. 51 mesiobuccal canals were randomly divided into 3 groups of 17 each. After instrumentation using the allocated file system i.e. Group 1 - Manual K files; Group 2 - Kedo SG Blue and Group 3 - Kedo S Square rotary files, CBCT images of the specimens were obtained. The remaining root dentin thickness was obtained using measurements of pre-and post-instrumentation CBCT. Data was analysed using paired t-test for intragroup comparisons. Intergroup comparison of RRDT was done using ANOVA.

**Results:**

The Remaining Root Dentin Thickness (RRDT) after instrumentation did not differ significantly among the three study groups at the three predetermined levels (*p* > 0.05).

**Conclusion:**

Kedo S Square and Kedo SG Blue file systems showed comparable effectiveness to the manual K file system in the instrumentation of mesiobuccal canals of primary molars.

## Background

Pulpectomy is the therapeutic choice to maintain the primary teeth with irreversibly damaged pulp. Adequate chemomechanical preparation of the canal is challenging to achieve owing to the anatomical complexity of the primary tooth root canals, which are extremely rich in anastomoses and auxiliary canals [1]. The lesser thickness of radicular dentin, inconsistent location of the apical foramen, and zones of physiologic resorption make chemomechanical preparation difficult [2]. 

There is a direct relationship between the root dentin thickness and the strength of the root. Excessive dentin removal can increase tooth fragility and the risk of perforation, zipping, canal transportation, and ledging. Microcracks can cause vertical fractures and eventually tooth loss. Additionally, pericervical dentin thickness is important in terms of the risk of crown-root fracture under chewing forces, the success of the restoration, and therefore the prognosis of the tooth and the treatment. Thus, the preparation must be performed in a manner that does not compromise the fracture resistance of the roots [3, 4]. 

The stainless steel hand files used for cleaning and shaping the root canals have limited flexibility, and their constant taper prolongs the treatment time, increases the risk of ledge formation, perforations, dentine compaction, instrument breakage, and inadequate root canal cleaning [1, 2]. The introduction of Nickel Titanium (NiTi) in the early 1990s provided respite from the limitations. Buchanan in 2000 introduced the concept of variable tapered root canal preparation to create a wider coronal third, which facilitated the movement of irrigants, and a smaller apical diameter that reduced the extrusion of debris. NiTi file systems with variable taper reduced the instrumentation time while their superior flexibility enabled the preservation of the original course of the root canal. This resulted in increased patient comfort and lowered the risk of flare-ups, as demonstrated in various in vitro and clinical trials done in permanent teeth [5]. 

Rotary systems designed for permanent teeth showed an increased incidence of instrument separation and perforations towards the danger zone of the root when used for the instrumentation of primary teeth [6, 7]. Thus, there was a perceived need for files with a modified taper, the proper length, and the proper tip size that were designed specifically for primary teeth [8]. 

Subsequently, the development of rotary file systems exclusively designed for primary teeth dramatically transformed the field of pediatric endodontics. The Kedo file system, designed and manufactured in 2016 by Reeganz Dental Care, Private Limited, India, includes five generations of files, the properties of which were improved over the generations. These 6 generations are known respectively as Kedo-S, Kedo-SG, Kedo-SG Blue, Kedo-S Square, Kedo-S Plus and Kedo Nano Plus [9, 10]. 

Kedo-SG Blue file system is a third-generation, 3-file system comprising D1, E1, and U1 Ni-Ti files. These heat-treated, titanium oxide-coated files have a variably variable (VV) taper and varying tip diameters (D1: 0.25, E1: 0.30, U1: 0.40) and a triangular cross-section throughout its length (12 mm). Their supreme flexibility prevents the accidental fracture of files in the intricate root canals of primary teeth [5, 10]. Better obturation of root canals and less apical extrusion of debris have been reported with the use of Kedo SG Blue file system [11]. 

Kedo S Square is the fourth-generation single file system, consisting of two files: one for anterior teeth (U1, with a tip diameter of 0.28, 4–8% taper) and one for posterior teeth (P1, with a tip diameter of 0.38, 6–8% taper). This Ni-Ti file is titanium oxide-coated and heat-treated, which adds to its flexibility. Dual cross-sections, triangular at the apical (5 mm) and tear drop at the coronal (7 mm) and variably variable taper correspond to the root canal morphology of primary teeth, which helps to achieve good tapered preparations by preserving the remaining root dentin [10, 12]. 

The manufacturers of Kedo range of specialized pediatric rotary files have been developed to overcome the limitations manual file systems thus enhancing their compatibility with primary root canal anatomy [10]. The efficacy of root canal instrumentation systems is studied using various parameters and different techniques. In this context, an important parameter to be evaluated is the amount of dentin remaining in the root canals after instrumentation.

Researchers have used numerous diagnostic methods, like conventional radiography, digital radiography, computed tomography (CT), micro-computed tomography (Micro CT), and cone-beam computed tomography to assess remaining root dentin thickness with different file systems. CBCT is a non-invasive, cost-effective technique with rapid image acquisition and reconstruction that provides a high-resolution three-dimensional visualisation of the intricacies of the root canal system [12]. 

This in-vitro study aimed to compare and evaluate the outcome of instrumentation with manual (K-file) and rotary (Kedo SG Blue and Kedo S Square) files, focusing on the remaining root dentin thickness of primary molar teeth using CBCT.

## Methods

This study was conducted in the Department of Paediatric and Preventive Dentistry in collaboration with the Department of Oral Medicine and Radiology, Manipal College of Dental Sciences, Manipal, Manipal Academy of Higher Education according to the stipulations of the Helsinki Declaration (2013) and previously approved by the Kasturba Medical College and Kasturba Hospital Institutional Ethics Committee under protocol number (IEC – 742/2020).

This in vitro study included 3 groups of seventeen mesiobuccal root canals each, making a total of 51 samples. In this single-blinded investigation, the investigator analysing the pre- and post-instrumentation CBCT images was blinded to the file systems used for the instrumentation of the root canals.

### Sample size calculation

Based on the values of previous research by Sharma et al. [13], the effect size of 0.435 was obtained. Substituting this value at 80% power and 95% confidence interval, the estimated sample size was 51.

### Specimen selection

Primary molar teeth used for the study were sourced from the out patients who reported to the Department of Pediatric and Preventive Dentistry for extraction either due to questionable prognosis for restoration, orthodontic reasons or refusal to undergo pulpectomy. Mandibular and maxillary first and second primary molars of patients between the ages of 3–8 years were collected as per the following inclusion and exclusion criteria: Sound or carious primary molar teeth with a minimum 7 mm root length measured from the cementoenamel junction (CEJ) were included. Teeth with less than 7 mm of root length measured from CEJ, teeth showing internal or external root resorption and caries perforating the furcation, with extreme root curvatures and teeth that show the presence of mesiobuccal 2 (MB2) in CBCT were excluded. The extracted teeth were cleaned of the remaining soft tissue, visible blood and debris using ultrasonic scalers and preserved in 0.1% thymol solution (KM chemicals, Mangalore, India) until further use.

### Specimen preparation

Endodontic access was obtained with a sterile access opening bur (Endo access bur, DENTSPLY, MAILLEFER), and the access cavity was refined using an Endo-Z bur (Endo access bur, DENTSPLY, MAILLEFER) to obtain straight-line access. The canals were located using a #10 K-file (Mani K files, Mani Inc., Tochigi, Japan) and the pulp chamber was irrigated with 1% sodium hypochlorite for 2 min. Any residual pulp tissue was extirpated using a barbed broach (#10, Mani Inc., Tochigi, Japan). A #10 K file with a stopper was introduced into the root canal until it was just discernible at the apical foramen to establish the working length. It was decided that the working length would be 1 mm shorter than the initial length.

### Specimen mounting and cone beam computed tomography

**Fig. 1:**
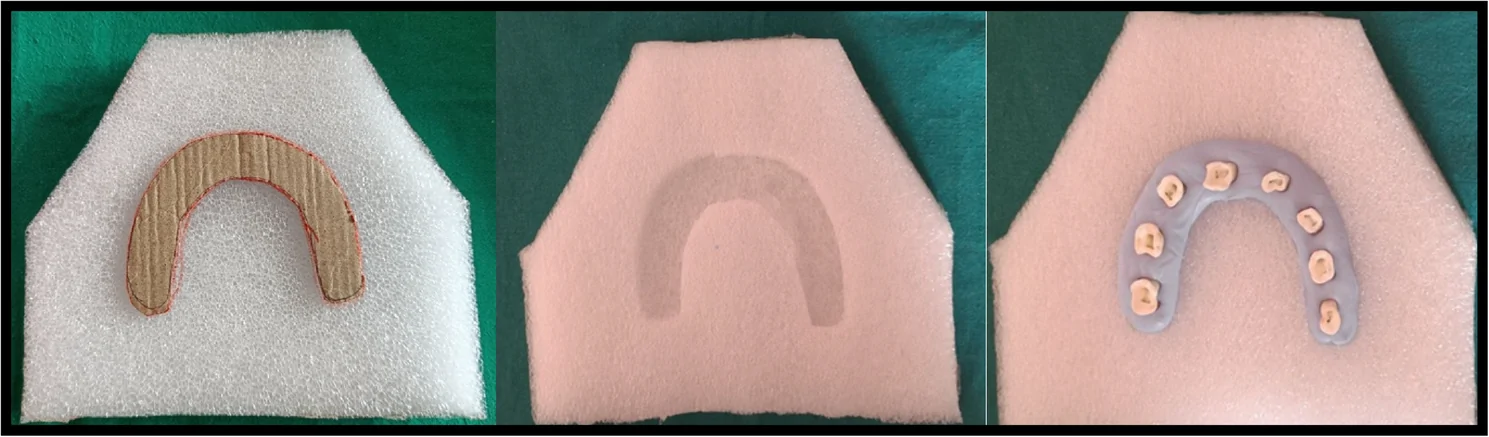
Indexing of foam sheet to accommodate impression material and sample teeth mounted for CBCT scanning

**Fig. 2:**
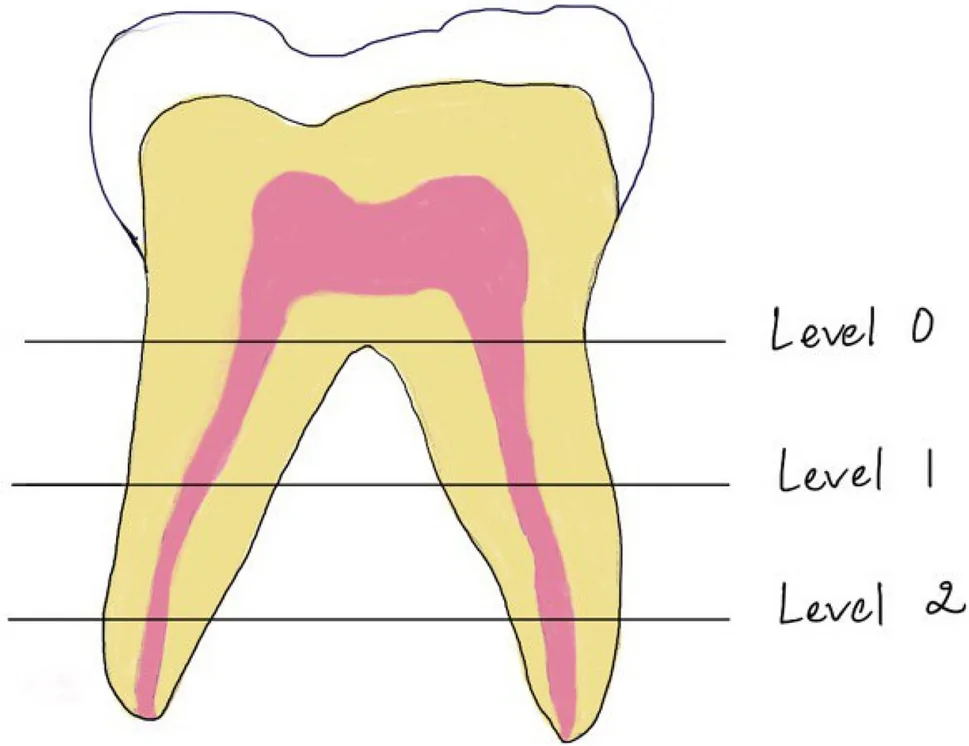
Schematic representation of the 3 levels at which root dentin thickness was measured in the study (Radhika et al., 2017 [14]). Level 0 – At the appearance of separation of canals just below the furcation. Level 1–2 mm below level 0. Level 2–4 mm below level 0

Sample teeth were mounted with roots embedded in the custom-made arch-shaped indices of high-viscosity hydrophilic vinyl polysiloxane impression material (Aquasil soft putty impression material, Dentsply). A polystyrene plastic foam sheet (Stylelabs EPE Foam, India) was used as a base for mounting and stabilizing the teeth in position to ensure standardization and reproducibility of images by the CBCT. Surface of the sample teeth was arranged such that it faced the inside of the arch in the labio-lingual direction (Fig. 1). The following exposure parameters were used: Scan time: 26.9 s, voxel size: 0.125 mm and beam diameter: 16 cm × 4 cm. The images were analysed using In Vivo Dental Anatomage software (Invivo dental, Anatomage inc., Osteoid Inc.,). The obtained DICOM (Digital Imaging and Communications in Medicine) images were viewed in the axial section and subjected to measurement of the area at three levels as illustrated in (Fig. 2).

The samples were subjected to Cone Beam Computed Tomography. The images were acquired on an i-CAT (i-CAT imaging system, Imaging Science International, Hatfield, PA, USA) at 3 predetermined levels (Fig. 2).

### Random allocation and pre-instrumentation measurement of root dentin thickness

After the pre-operative CBCT, the samples were randomized using a table of random numbers. 51 teeth were randomized using a 1:1:1 equal allocation ratio and a block size of three.

**Fig. 3:**
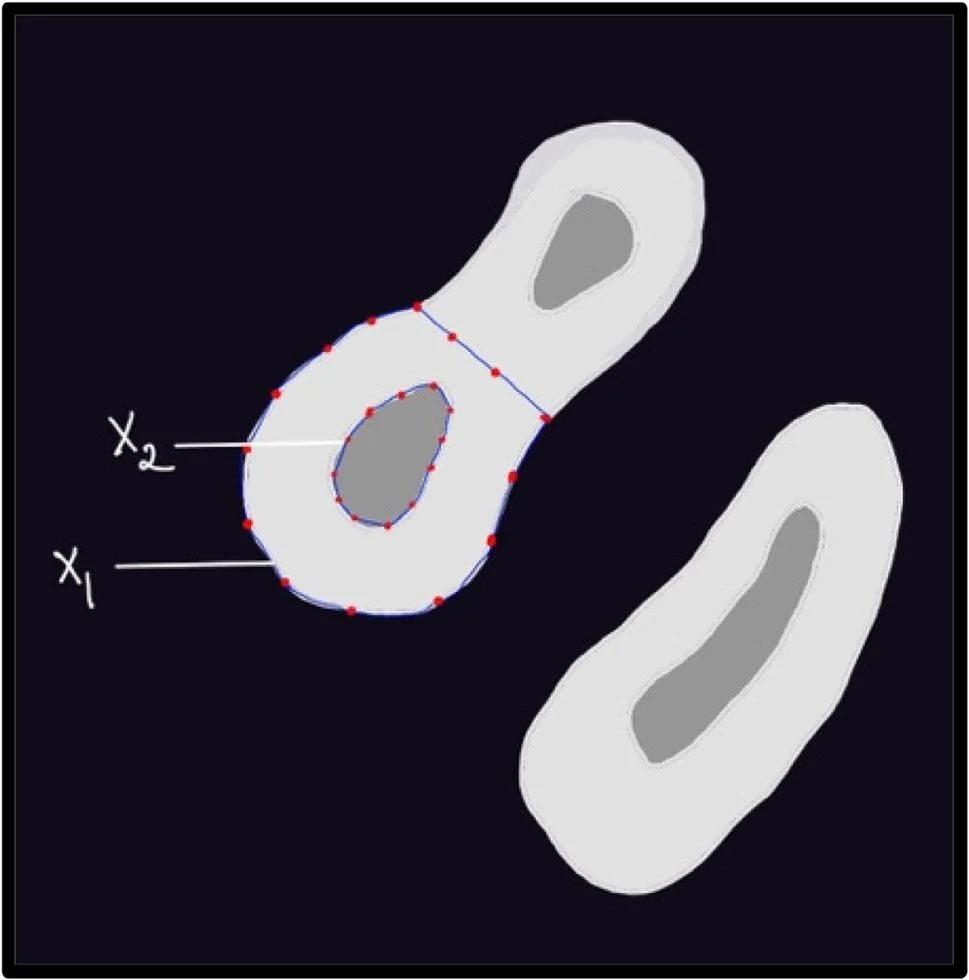
Schematic representation and CBCT image of inner and outer dentin area demarcation for the estimation of the root dentin thickness in the mesial root of a mandibular molar

An area marking tool from the In Vivo Dental Anatomage software (Invivo dental, Anatomage inc., Osteoid Inc.,) was employed to mark the points at the outer border of the root (X1) and the outer border of pulp space (X2). The area obtained in mm^2^ was subtracted (X1 – X2) to acquire the root dentin thickness at the 3 different predetermined levels (Fig. 3).

The demarcation at the centre of the root for the mandibular molar roots was decided by visual inspection of the axial section by the investigators to divide the entire root with the two root canals equally into two parts. This challenge for radiographic measurement was identified at the commencement of the study and the two expert oral and maxillofacial radiologists were trained for the measurements at different levels for standardisation. The inter- observer and intra-observer reliability was analysed for 10% of the measurements which showed excellent correlation (> 0.9).

### Chemo mechanical preparation of root canals

- *Group 1 (K-file*, Mani K files, Mani Inc., Tochigi, Japan; 0.02 taper**)**: The canals were instrumented using K-files in a reciprocating back-and-forth motion to the #40 K-file using the step-back technique with periodic recapitulation done using the #15 K file [14].
- *Group 2 (Kedo-SG Blue*** (**Reeganz Dental Care Pvt. Ltd. India**)**: A #15 K-file was used in the canals in a reciprocating back-and-forth motion passively till the working length. Since mesiobuccal canals were included, only D1 rotary file was used for instrumentation of this canal in maxillary and mandibular teeth in a lateral brushing motion following the crown-down technique as recommended by the manufacturer [9].
- *Group 3 (Kedo-S Square*** (**Reeganz Dental Care Pvt. Ltd. India**)**: Following the manufacturer’s recommendation a #15 K-file was used in the canals in a reciprocating back-and-forth motion passively till the working length. (P1) Kedo S Square rotary file was used in a lateral brushing motion till the working length following the crown down technique as recommended by the manufacturer [9].

The rotary files were used for the instrumentation of canals with an endomotor (COLTENE ENDO, CanalPro CL2 (LED) optic Cordless, Coltene Whaledent Pvt. Ltd) at 300 rpm and 2.4 Ncm torque for a minimum of 1–2 times in the root canals as recommended by the manufacturer. The hand and rotary files were examined for distortion of flutes using a handheld magnifying lens each time after withdrawal from the canal. Such distorted files were discarded. Each file was used only for the instrumentation of 5 samples in accordance with the guidelines provided by the manufacturer.

The root canal irrigation was done using BD Emerald Syringe and needle (BD Medical (S) Ptc Ltd, Singapore) with 1% sodium hypochlorite (MEDILISE Chemicals, Kannur, Kerala, India) followed by 0.9% w/v saline (Fresenius Kabi India Pvt. Ltd.) after the use of each file. During the chemomechanical preparation, the files were lubricated with 17% EDTA (Endoprep-RC, Anabond Stedman Pharma Research (P) Ltd, India) before the insertion into the canals. Finally, sterile paper points were used to dry the canals. To avoid bias in preparation caused by operator fatigue, the primary investigator instrumented up to six teeth at once [15]. The samples were subjected to CBCT after chemo-mechanical preparation of root canals to obtain post-instrumentation measurements as mentioned previously by the experienced, radiologist who was blinded to the allocation and calibrated to the procedure; thus the values of the remaining root dentin thickness were obtained.

**Fig. 4:**
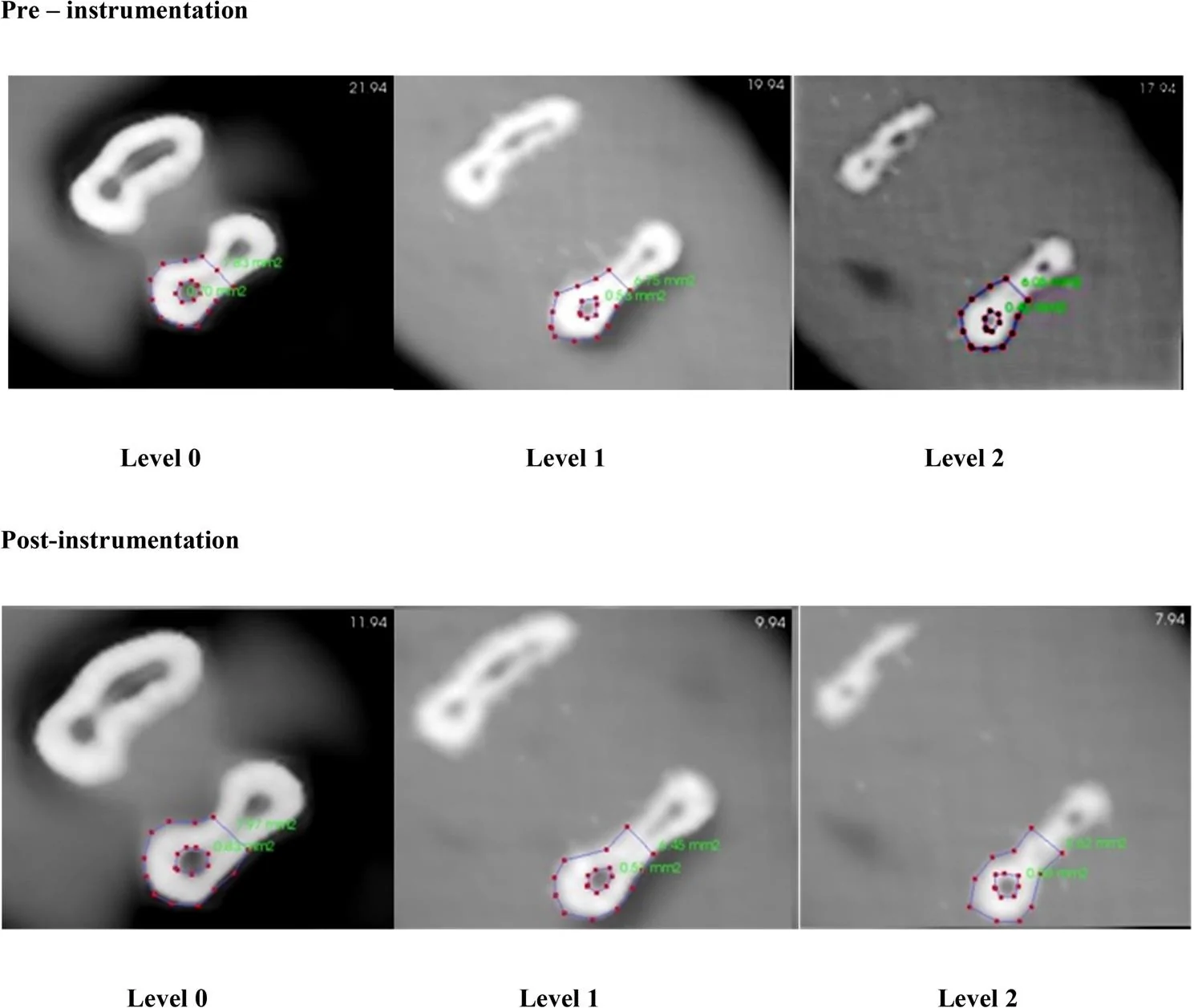
Pre and post-instrumentation CBCT images of a representative sample from group 1 (K-file)

**Fig. 5:**
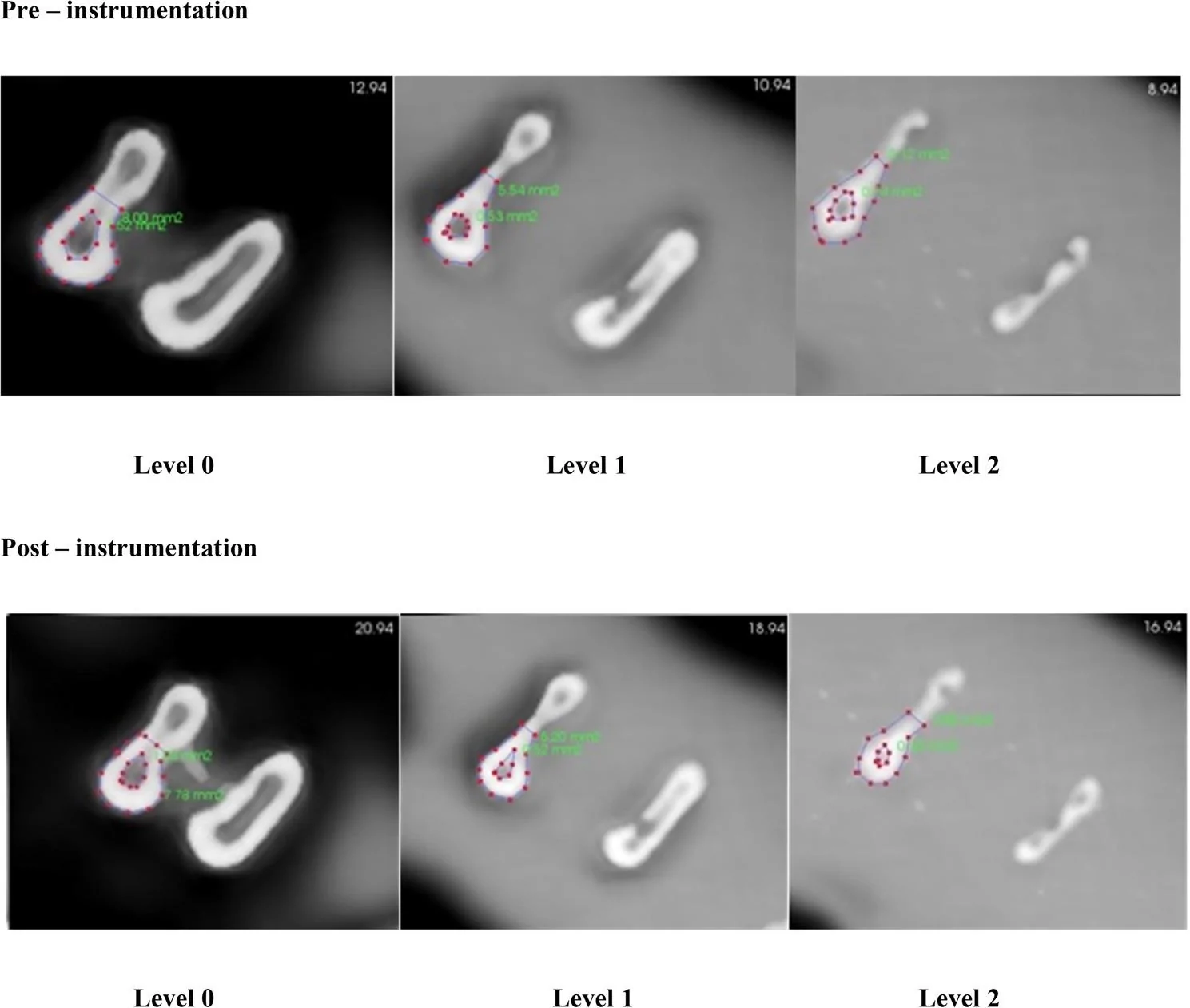
Pre and post-instrumentation CBCT images of a representative sample from group 2 (Kedo SG Blue file)

**Fig. 6:**
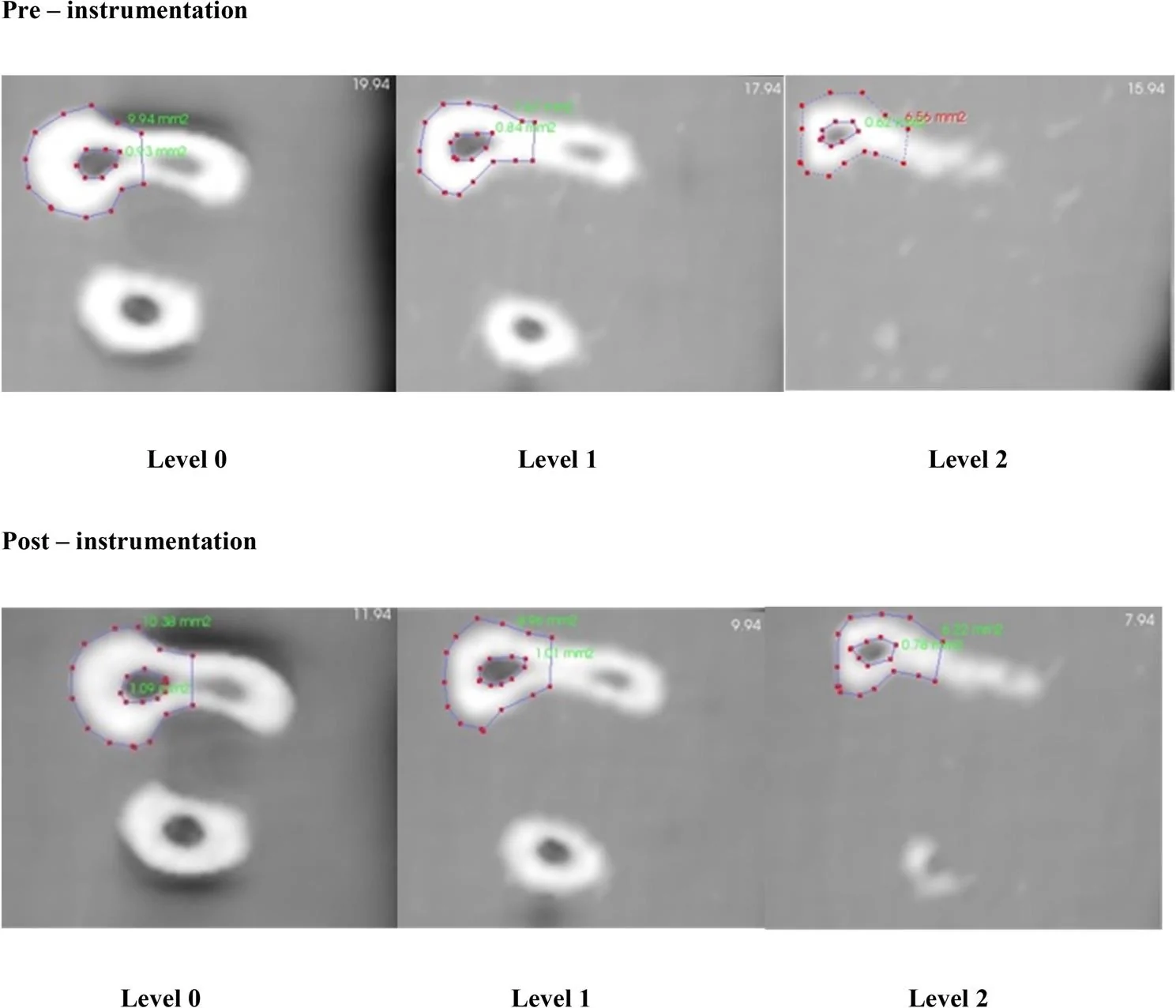
Pre and post-instrumentation CBCT images of a representative sample from group 3 (Kedo S Square file)

Pre and post-instrumentation CBCT images of representative samples from the hand K file, Kedo SG Blue and Kedo S Square rotary file groups are given in Figs. 4, 5 and 6, respectively. 

Comparison of pre and post instrumentation CBCT images did not reveal any evidence of perforation/ canal transportation in the study samples.

### Statistical analysis

Data were analyzed using the statistical package MINITAB 19.0 (Minitab, LLC, Pennsylvania, USA), and the level of significance was set at *p* < 0.05 (95% confidence). Descriptive statistics were performed to assess the mean and standard deviation of the respective groups. The normality of the data was assessed using the Shapiro-Wilkinson test. Inferential statistics to find out the difference between the groups was done using ANOVA (Analysis of Variance). The repeatability of the measurements was measured using Pearson’s correlation coefficient analysis.

## Results

The sample teeth comprised 12 maxillary and 39 mandibular molar teeth. At baseline, the intergroup comparison of root dentin thickness across the study groups at the three pre-determined levels was statistically insignificant.

Table 1 shows the values of root dentin thickness pre- and post-instrumentation expressed as mean and standard deviation for all the study groups. Intragroup comparison using paired t-test showed p < 0.001 for the three file systems, suggesting effective dentin removal at three pre-determined levels (Table 1)

**Table 1:** Intragroup comparison of pre- and post-instrumentation root dentin thickness for samples of the three groups

Level		K file				Kedo SG Blue file						Kedo S Square file		
	Pre-instrumentation (Mean ± SD)	Post-instrumentation (Mean ± SD)	Mean difference (Percentage reduction)	*p*-value (t-value)	Pre-instrumentation (Mean ± SD)		Post- instrumentation (Mean ± SD)	Mean difference (Percentage reduction)	*p*-value (t-value)	Pre-instrumentation (Mean ± SD)	Post-instrumentation (Mean ± SD)		Mean difference (Percentage reduction)	*p*-value (t-value)
Level 0	7.86 ± 1.45	6.11 ± 1.17	1.75 ± 0.81 (22.4%)	< 0.001* (-8.8993)	7.8 ± 1.53		6.14 ± 1.56	1.66 ± 1.36 (21.3%)	< 0.001* (-5.0291)	7.86 ± 1.68	6.19 ± 1.16		1.67 ± 1.26	< 0.001* (-5.49)
Level 1	5.66 ± 1.39	4.96 ± 1.4	0.69 ± 0.63 (12.3%)	< 0.001* (-4.5022)	5.75 ± 1.35		4.78 ± 1.2	0.97 ± 0.89 (16.8%)	< 0.001* (-4.4612)	5.99 ± 0.92	5.15 ± 0.77		0.84 ± 0.71	< 0.001* (-4.86)
Level 2	3.96 ± 1.44	3.16 ± 1.22	0.8 ± 0.64 (20.2%)	< 0.001* (-5.144)	4.21 ± 1.07		3.34 ± 0.81	0.87 ± 0.76 (20.6%)	< 0.001* (-4.7132)	3.95 ± 0.79	3.17 ± 1.15		0.6 ± 0.74	< 0.001* (-3.3633)

*statistically significant by paired t test; Values expressed as mm^2^

The mean difference pre and post instrumentation at level 0 was highest in the K file group and least in Kedo SG blue group. At level 1the mean difference was highest in Kedo SG blue group followed by Kedo s square group and least in K file group. The mean difference in level 2 was greatest in Kedo SG group followed by K file group and least in Kedo S square group.

Table 2 shows the remaining root dentin thickness post instrumentation across the study groups at three predetermined levels. ANOVA showed non-significant difference between the groups (Level 0 - *p* = 0.9856; Level 1- p = o.6582; Level 2 - *p* = 0.8556) indicating comparable effectiveness of the file systems in dentin removal.

**Table 2:** Intergroup comparison of post-instrumentation root dentin thickness across the three study groups

Level	K file	Kedo SG Blue	Kedo S Square	*p*-value
Level 0	6.11 ± 1.17	6.14 ± 1.56	6.19 ± 1.16	0.9856
Level 1	4.96 ± 1.4	4.78 ± 1.2	5.15 ± 0.77	0.6582
Level 2	3.16 ± 1.22	3.34 ± 0.81	3.17 ± 1.15	0.8556

*p* > 0.05 – statistically insignificant using ANOVA; Values expressed as mm^2^

## Discussion

Three factors determining the success of pulpectomy are optimal access to the pulp chamber and root canals, appropriate cleaning and shaping and effective three-dimensional sealing [16, 17]. Chemomechanical preparation is the most crucial step of pulpectomy owing to the complexity of the canal anatomy, dynamic alterations at the apex, closeness to the developing tooth bud, along with perceived difficulty in behaviour management and isolation [2, 18]. 

The use of stainless-steel files for root canal preparation has long been considered the “gold standard”. These small tapered files lengthen the preparation time; their stiffness limits the apical enlargement, which hinders effective irrigation and results in the formation of ledges and perforations [18]. The introduction of Nickel-Titanium (NiTi) based rotary file systems has resulted in a quantum leap in the mechanical preparation of the root canals. These larger tapered files create funnel-shaped canals which ease irrigation, reduce apical extrusion of debris, and result in optimal obturation along with enhanced patient comfort due to less time taken and minimal post-operative pain [19–21]. 

Several authors have reported difficulty in the instrumentation of ribbon-shaped canals of primary molars using rotary systems specifically designed for permanent teeth. Increased incidences of instrument fractures were also reported [6, 8]. A modified protocol for the use of these files in primary teeth was suggested by Musale et al. [15] to overcome these drawbacks. Subsequently, instruments designed to suit the morphology of primary root canals have been developed and studied.

Kedo S is an exclusive pediatric file system designed with a variably variable taper to suit primary root canal anatomy and aids in cleaning and shaping the canals while resisting cyclic fatigue [11]. Its efficacy has been assessed in the cleaning of root canals [22], centering ability and canal transportation [23], the ability to produce a tapered shape of the root canal, the amount of dentin removed during instrumentation [18] and apical extrusion of debris [11]. Literature shows sparse studies on the newly introduced Kedo S Square file system in primary molars. Therefore, this study assessed the root dentin thickness remaining after instrumentation using Kedo SG Blue and Kedo S square file systems in comparison to the “gold standard” K file system.

In vitro assessment of the efficacy of root canal instrumentation can be performed on resin blocks or extracted human teeth [17, 18]. While resin blocks provide the ideal comparability, extracted human teeth pose constraints of non-uniform apical foramen, compatibility of the apex with a particular instrument size, and the angle of curvature [24]. Since the focus of this study was to mimic the clinical setting as closely as possible, extracted human primary teeth were used. Root length of sample teeth was standardized to a minimum of 7 mm from the CEJ in this study which was considered based on prior knowledge about the average lengths of mesiobuccal roots of maxillary molar (1st molar – 10 mm; 2nd molar – 7.8 mm) and mesial root of mandibular molar (1st molar – 9.8 mm; 2nd molar – 11.3 mm) [25]. Similar criteria were used in previous studies [15, 26]. Primary molars exhibit considerable variations in their root canal morphology, where the greatest variation is seen in the distal canals of mandibular molars with the presence of more than one canal [27]. To have better comparability, only the mesiobuccal canals of primary maxillary and mandibular molars were considered for this study. Studies have reported various methods of root dentin measurement to assess the shaping ability of an instrument, using a variety of techniques like assessing the thickness of the dentin remaining in terms of volume and area [13], canal transportation and centering ability [16], and determining untouched canal surfaces [1]. The Remaining Root Dentin Thickness (RRDT) is the measure of the mechanical limits of instrumentation to widen the canals to a predetermined level without considerably weakening the dentinal walls. The thickness of dentin remaining after instrumentation is positively correlated to the aggressiveness of the instrument, and there are no guidelines that specify how much dentin can be removed during the preparation of the canals [26]. Since the strength of the root is directly proportional to the root dentin thickness, excessive dentin removal can render the tooth fragile with greater risk for vertical fracture and eventual tooth loss. Particularly, the preservation of pericervical dentin thickness will improve the success of the treatment, and therefore the prognosis of the tooth [3, 4]. Manker et al. [2], suggest retaining a minimum of 1 mm of sound radicular dentin after the canal preparation. Since the amount of dentin in root canals of primary molars is not uniform due to the flat and ribbon-shaped canals and root resorption, selective filing is advocated when using conventional hand files to avoid inadvertent perforations [28]. NiTi rotary file systems with variable taper remove less dentin compared to hand files and are advantageous in primary root canals, as demonstrated by various studies [1, 12, 13]. Another factor to be considered is the rate of exfoliation of the pulpectomized primary teeth. It is reported that thin root canal walls remaining after aggressive removal of dentin result in faster exfoliation of the tooth [18, 26]. Hence, it was considered valid to conduct this investigation on primary molars using hand and rotary systems by evaluating the root dentin thickness pre- and post-instrumentation. Authors have studied and reported different ways of determining RRDT [6, 7, 27, 29, 30]. Some of these methods are destructive and do not provide 3D analysis of the specimens. CBCT, being a suitable, accessible and affordable alternative to Micro CT, was employed in this study to assess and compare the remaining root dentin thickness [12, 15–17]. Authors have suggested different ways for mounting the teeth for CBCT evaluation [16, 30, 31]. The mounting technique followed the study conducted by Abdelkafy et al., [24], which simulated the dental arch and enabled exposure of multiple teeth at a time, thus reducing the time and cost involved.

Different ways of measuring the remaining root dentin, which include the linear measurement from the pulp space to the outer border of dentin, [16] construction and superimposition of masks [7] area estimation using third-party software [13] and voxel measurements. [1] A pilot study was conducted before the main study to understand the best-suited method to be used to measure dentin thickness. Since obtaining reproducible linear measurements proved to be difficult, it was decided to use an area marking tool. This study used Invivo software to measure the tooth at 3 different levels in axial sections of 0.2 mm slice thickness. So the errors of manually measuring the length of the roots at three levels from the root apex were avoided.

In this study, the comparison of root dentin thickness preoperatively showed statistical insignificance, thus ensuring the comparability of samples at baseline. Post-instrumentation CBCT assessment showed effective dentin remaining at all the predetermined levels in both rotary and manual systems, which was statistically insignificant. This result was consistent with the results reported by Prabhakar et al., [32] and Ghahramani et al., [17] who found no discernible difference in the dentin removed between manual and other rotary systems used in primary teeth. On the contrary, studies by Poornima et al., [33] found a significant increase in dentin removal by the K file compared to rotary files. Musale et al., [26] and Esentürk et al., [1] found a significant increase in dentin removal by the different rotary systems compared to the K files.

In the present study, the difference in the remaining root dentin thickness was satistically insignificant across the three study groups. However, the values of mean difference at the three levels indicate that more pericervical dentin was preserved in the Kedo SG blue and and Kedo S square file systems compared to K files, and this observation is of clinical significance. Similar results were observed in previous studies, where a significant amount of dentin was removed by the K file system at the cervical region of the root canal compared to other rotary file systems. [16, 18, 26] These findings may be attributed to the design of the K file. These less flexible stainless steel files have a 16 mm cutting blade with a cutting tip, a square cross-section, and a fixed 2% taper through which they laterally press against the dentinal walls to remove dentin. [26] Contrary to the above result Barasuol et al., [30] showed significant dentin removal in the apical third of the canal with K files and attributed it to their lower flexibility.

Kedo SG blue file system showed a decreased amount dentin remaining at the middle and apical levels. This result is in concurrence with studies by Musale et al., [26] and Sharma et al., [13] which could be attributed to the increased taper of rotary NiTi files compared to the fixed taper of hand files. The Kedo-S Square file system used in this study presented a greater conservation of dentin. This is in concurrence with the results by Mohamed et al., [12] These findings may be attributed to the properties of the Kedo rotary file system. Kedo SG Blue (D1) files have a variably variable taper and a triangular cross-section along the length of the file resulting in superior flexibility of the file. [12] Kedo S Square files are titanium oxide-coated, heat-treated files, having a non-cutting tip and a variably variable taper. They have a dual cross-section, triangular in the apical 5 mm and teardrop in the coronal region. This creates lesser apical and greater coronal preparation of the root canals permitting the flow of obturating material. [10, 12]

One of the limitations of our study was the possibility of subjective error in measurement of the remaining root dentin thickness. Minimal measurement differences could have arised due to differences in radiologists’ skill, or technique adopted for selection of the sections to measure dentin thickness at three different levels. However this was taken care by prior calibration of the measurement techniques by expert radiologists of over 10 years experience. Level of placement of the mounted samples on the machine and the optimal parameters for image acquisition were standardized and customized with pilot investigation. The final parameters used in the study were specific to the CBCT machine used in our setting. However, the reproducibility of the same parameters with another machine needs evaluation.

Further, the present study included only mesiobuccal canals of primary molars. Diversifying this experiment to include multiple canal types with varying morphology will strengthen evidence on the efficacy of these file systems.

The results of this in vitro study cannot be directly extrapolated to the clinical scenario without supporting clinical studies. Further research can focus on in vivo validation studies comparing various contemporary rotary systems specifically designed for use in primary teeth. Such investigations will not only provide an in depth understanding of rotary file systems, but will also underscore the relevance of structured education and training in adopting rotary systems into pediatric dental practice.

## Conclusions

Within the limitations of the present invitro study, the tested rotary files showed comparable effectiveness to manual K files in terms of remaining root dentin thickness after instrumentation. Although differences in the remaining root dentin thickness were statistically insignificant across the study groups, the use of manual files showed the least remaining dentin in the cervical aspect of the root compared to the rotary file systems .

## Data Availability

The data used to support the findings of this study are available from the corresponding author upon request.

## Abbreviations

CBCT: Cone Beam Computed Tomography
Ni: Ti-Nickel Titanium
Kedo S: Kids Endodontic Shaper
EDTA: Ethylenediaminetetraacetic acid
rpm: Rotations Per Minute
RRDT: Remaining root dentin thickness
ANOVA: Analysis of Variance
DICOM: Digital Imaging and Communications in Medicine

## References

[1] Esentürk G, Akkas E, Cubukcu E, Nagas E, Uyanik O, Cehreli ZC. A micro-computed tomographic assessment of root canal preparation with conventional and different rotary files in primary teeth and young permanent teeth. Int J Paediatr Dent. 2020;30(2):202–8. 10.1111/ipd.12587. 31651057

[2] Manker A, Solanki M, Tripathi A, Jain ML. Biomechanical preparation in primary molars using manual and three niti instruments: a cone-beam-computed tomographic in vitro study. Eur Arch Paediatr Dent. 2020;21(2):203–13. 10.1007/s40368-019-00474-0. 31489569

[3] Eren I, Sezer B. Comparative evaluation of the remaining dentin volume following instrumentation with rotary, reciprocating, and hand files during root canal treatment in primary molars: an ex vivo study. J Dent Sci. 2024;19(4):2126–34. 10.1016/j.jds.2024.04.003. 39347077 PMC11437276

[4] Nisar P, Katge F, Bhanushali P, Deshpande S, Poojari M, Shetty S. Comparative in vitro evaluation of remaining dentine thickness following instrumentation with hand and rotary endodontic files during pulpectomy in primary molars: a systematic review. Eur Archives Pediatr Dentistry. 2023;24(1):15–32. 10.1007/s40368-022-00760-4. 36319891

[5] Priyadarshini P, Jeevanandan G, Govindaraju L, Subramanian EM. Clinical evaluation of instrumentation time and quality of obturation using paediatric hand and rotary file systems with conventional hand K-files for pulpectomy in primary mandibular molars: a double-blinded randomized controlled trial. Eur Arch Paediatr Dent. 2020;21(6):693–701. 10.1007/s40368-020-00518-w. 32185634

[6] Nagaratna PJ, Shashikiran ND, Subbareddy VV. In vitro comparison of niti rotary instruments and stainless steel hand instruments in root canal preparations of the primary and permanent molar. J Indian Soc Pedod Prev Dent. 2006;24(4):186. 10.4103/0970-4388.28075. 17183182

[7] Hidalgo LR, Silva LA, Leoni GB, Mazzi-Chaves JF, Carvalho EE, Consolaro A, Sousa-Neto MD. Mechanical preparation showed superior shaping ability than manual technique in primary molars-a micro-computed tomography study. Braz Dent J. 2017;28:453–60. 10.1590/0103-6440201601552. 29160397

[8] Kuo C, Wang Y, Chang H, et al. Application of Ni–Ti rotary files for pulpectomy in primary molars. J Dent Sci. 2006;1:10–5. 10.1007/s40368-018-0356-6.

[9] Manufacturer’s brochure on Kedo S file systems. (Reeganz Dental Care Pvt. Ltd, India). https://kedodental.com/catalogue/.

[10] Suresh B, Jeevanandan G, Ravindran V. Revolutionizing Pulpectomy: An Observational Overview of Multigenerational Kedo Rotary File Systems in Primary Molars. Cureus. 2024;16(7). 10.7759/cureus.65147. PMC11341114 39176360

[11] Peedikayil FC, Avaneethram A, Kottayi S, Premkumar CT, Aparna T, Aravind A. Comparative Evaluation of Apical Extrusion of Debris Using Hand and Rotary Assisted Instrumentation in Primary Single Rooted Teeth: An In-vitro Study. J Clin Diagn Res. 2022;16(9). 10.7860/JCDR/2022/55352.16842.

[12] Mohamed RH, Abdelrahman AM, Sharaf AA. Evaluation of rotary file system (Kedo-S-Square) in root canal preparation of primary anterior teeth using cone beam computed tomography (CBCT)-in vitro study. BMC Oral Health. 2022;22(1):1–0. 10.1186/s12903-021-02021-0. 35042489 PMC8764846

[13] Sharma P, Kurthukoti AJ, Sebastian A, Mallikarjun SB. Comparative evaluation of change in root canal shape and area in primary molars using ProTaper and K3 rotary systems: In vitro study. J Indian Soc Pedod Prev Dent. 2021;39(4):392. 10.4103/jisppd.jisppd_30_21. 35102964

[14] Radhika E, Reddy ER, Rani ST, Kumar LV, Manjula M, Mohan TA. Cone beam computed tomography evaluation of hand nickel-titanium K-files and rotary system in primary teeth. Pediatr Dent. 2017;39(4):319–23. (PMID: 29122074). 29122074

[15] Musale PK, Mujawar SA. Evaluation of the efficacy of rotary vs. hand files in root canal preparation of primary teeth in vitro using CBCT. Eur Arch Paediatr Dent. 2014;15(2):113–20. 10.1007/s40368-013-0072-1. 23893606

[16] Shaikh SM, Goswami M. Evaluation of the effect of different root canal preparation techniques in primary teeth using CBCT. J Clin Pediatr Dent. 2018;42(4):250–5. 10.17796/1053-4628-42.4.2. 29750624

[17] Ghahramani Y, Mohammadi N, Zangooei-Booshehri M, Shirdel S. Comparing the amount of removed dentin thickness in root canal treated primary molar teeth using different instrumentation techniques: an in-vitro study using CBCT. Eur Arch Paediatr Dent. 2022;23(2):255–60. 10.1007/s40368-021-00662-x. 34494202

[18] Seema T, Ahammed H, Parul S, Cheranjeevi J. Comparative evaluation of dentin removal and taper of root canal preparation of hand K file, ProTaper rotary file, and kedo s rotary file in primary molars using cone-beam computed tomography. Int J Clin Pediatr Dent. 2020;13(4):332. 10.5005/jp-journals-10005-1787. 33149404 PMC7586477

[19] Jeevanandan G, Govindaraju L. Clinical comparison of Kedo-S paediatric rotary files vs manual instrumentation for root canal preparation in primary molars: a double-blinded randomized clinical trial. Eur Arch Paediatr Dent. 2018;19(4):273–8. 10.1007/s40368-018-0356-6. 30003514

[20] Panchal V, Jeevanandan G, Subramanian EM. Comparison of postoperative pain after root canal instrumentation with hand K-files, H-files and rotary Kedo-S files in primary teeth: a randomized clinical trial. Eur Arch Paediatr Dent. 2019;20(5):467–72. 10.1007/s40368-019-00429-5. 30864090

[21] Elheeny AA, Abdelmotelb MA. Postoperative pain after primary molar pulpectomy using rotary or reciprocating single files: A superior, parallel, randomized clinical trial. Int J Paediatr Dent. 2022 May;5. 10.1111/ipd.12959. 35152509

[22] Tofangchiha M, Ebrahimi A, Adel M, Kermani F, Mohammadi N, Reda R, Testarelli L. In vitro evaluation of Kedo-S and RaCe rotary files compared to hand files in preparing the root canals of primary molar teeth. Front Biosci (Elite Ed). 2022;14(2):14. 10.31083/j.fbe1402014. 35730455

[23] Waly A, Yamany I, Abbas H, Alsairafi MA, Bazzaz RF, Bogari D, Alhazzazi T. Comparison of two pediatric rotary file systems and hand instrumentation in primary molar: An ex vivo cone-beam computed tomographic study. Niger J Clin Pract. 2021;24(10):1492–. 10.4103/njcp.njcp_563_20. 34657015

[24] Abdelkafy H, Eldehna AM, Salem NA. Canal Transportation and Centering Ratio of Paediatric vs Regular Files in Primary Teeth. Int Dent J l. 2022 Oct;11. 10.1016/j.identj.2022.09.003. PMC10213759 36241464

[25] Nelson SJ. Wheeler's Dental Anatomy, Physiology and Occlusion-E-Book: Wheeler's Dental Anatomy, Physiology and Occlusion-E-Book. Elsevier Health Sciences; 2014.

[26] Musale PK, Jain KR, Kothare SS. Comparative assessment of dentin removal following hand and rotary instrumentation in primary molars using cone-beam computed tomography. J Indian Soc Pedod Prev Dent. 2019;37(1):80. 10.4103/jisppd.jisppd_210_18. 30804312

[27] Selvakumar H, Anandhan V, Thomas E, Swaminathan K, Vijayakumar R. Evaluation of canal transportation and centering ability of K 3 (0.02%) and K 3 (0.04%) with hand K files in primary teeth using spiral computed tomography. J Indian Soc Pedod Prev Dent. 2014;32(4):286. 10.4103/0970-4388.140943. 25231035

[28] Tandon S. Textbook of pedodontics. Paras Medical Publisher; 2009.

[29] Canoglu H, Tekcicek MU, Cehreli ZC. Comparison of conventional, rotary, and ultrasonic preparation, different final irrigation regimens, and 2 sealers in primary molar root canal therapy. Pediatr Dent. 2006;28(6):518–23. (PMID: 17249433). 17249433

[30] Barasuol JC, Alcalde MP, Bortoluzzi EA, Duarte MA, Cardoso M, Bolan M. Shaping ability of hand, rotary and reciprocating files in primary teeth: a micro-CT study in vitro. Eur Arch Paediatr Dent. 2021;22(2):195–201. 10.1007/s40368-020-00530-0. 32346833

[31] Khattab NM, Gomaa YF, Elheeny AA. Cone-beam computed tomography analysis of primary root canal transportation and dentin loss after instrumentation with two-pediatric rotary files. BMC Oral Health. 2022;22(1):1–8. 10.1186/s12903-022-02245-8. 35641977 PMC9153195

[32] Prabhakar AR, Yavagal C, Dixit K, Naik SV. Reciprocating vs rotary instrumentation in pediatric endodontics: cone beam computed tomographic analysis of deciduous root canals using two single-file systems. Int J Clin Pediatr Dent. 2016;9(1):45. 10.5005/jp-journals-10005-1332. 27274155 PMC4890062

[33] Poornima P, Disha P, Nagaveni NB, Roopa KB, Bharath KP, Neena IE. Volumetric analysis of hand and rotary root canal instrumentation and filling in primary teeth using Spiral Computed Tomography’–an in-vitro study. Int J Paediatr Dent. 2016;26(3):193–8. 10.1111/ipd.12180. 26146902

